# Impact of renal disease and comorbidities on mortality in hemodialysis patients with COVID-19: a multicenter experience from Germany

**DOI:** 10.1007/s40620-020-00828-8

**Published:** 2020-08-17

**Authors:** Maximilian Seidel, Bodo Hölzer, Heiner Appel, Nina Babel, Timm H. Westhoff, The COVID Dialysis Working Group, Thiemo Pfab, Jan Hörstrup, Andrea Uhle, Michael Frahnert, Michael Barenbrock, Eckhardt Büssemaker, Adrian Doevelaar, Felix S. Seibert, Frederic Bauer

**Affiliations:** 1 Medical Department I, University Hospital Marien Hospital Herne, Ruhr-University Bochum, Hölkeskampring 40, 44625 Herne, Germany; 2 Dialysezentrum Hamm, Hamm, Germany; 3 Center for Translational Medicine, University Hospital Marien Hospital Herne, Ruhr-University Bochum, Bochum, Germany

There is an increasing number of reports on the clinical course of Coronavirus disease 2019 (COVID-19) in hemodialysis patients [1–4].

On this background, we would like to report data on a large multicenter cohort of hemodialysis patients with COVID-19 in Germany, which demonstrates an inverse distribution of mild and severe courses compared to the general population. Through the analysis of the impact of underlying renal disease and cardiovascular comorbidities on adverse outcome, we could identify cardiorenal syndrome as an outstanding risk factor for death.

Since the first cases of pneumonia of unknown origin were reported in December 2019 in Wuhan, knowledge on SARS-CoV-2 and COVID-19 is rapidly expanding. Preexisting cardiovascular disease, diabetes, hypertension, and chronic kidney disease were identified as risk factors for severe disease and mortality [5–7]. Thus, the hemodialysis population may be at outstanding risk for a severe course of COVID-19. Moreover, hemodialysis centers are prone to SARS-CoV-2 transmission. Patients have to refer to the outpatient facility three times per week to undergo dialysis and are thereby limited in their ability to social distance. As expected, mortality rates were substantially higher than in the general population, ranging from 18.9 (Wuhan) to 52% (Lombardy) [1, 2].

Interestingly, the causes of death were frequently not directly related to pneumonia but to cardiovascular or cerebrovascular disease [1]. Although there was a shift to more critical courses of COVID-19, the spectrum of severity of COVID-19 was similar to the general population including several asymptomatic patients. It remains elusive which hemodialysis patients are at increased risk for adverse outcome. We would therefore like to add to the present knowledge by reporting on the clinical characterization of COVID-19 in hemodialysis patients in Germany and identifying risk factors for adverse outcome.

Five hemodialysis outpatient centers in Germany were contacted in April 2020 and asked to participate in the analysis. All of them agreed and provided clinical data of their hemodialysis patients with COVID-19 from February to April 2020. Data collection was performed by nephrologists from the outpatient centers and included both outpatient data and in-hospital data; analysis was centralized at a University Hospital (Ruhr-University Bochum). Data comprised course of the disease, stratified as "mild", "severe", "critical", or "fatal". The disease was considered as "mild" if it was successfully managed in an outpatient setting, "severe" if it needed hospitalization, "critical" in case of transfer to an intensive care unit, and "fatal" in case of death. Intensive care medicine was offered to all patients with a medical indication. Information on the following symptoms was retrieved: fever, cough, dyspnea, dysgeusia, anosmia, diarrhea. Information on cause of end-stage kidney disease (ESRD), preexisting comorbidities and immunosuppression was also obtained. Associations of underlying renal diseases and comorbidities with mortality were analyzed by Chi-squared tests and univariate analyses providing odds ratios (OR) and 95% confidence intervals (CI).

Fifty-six patients tested positive for SARS-CoV-2 by RT-PCR (SARS-CoV-2 RT-PCR Kit 1.0 from Altona Diagnostics, Hamburg) in either nasopharyngeal swab test or bronchoalveolar lavage. The overall number of patients on chronic hemodialysis in the five centers was 755 with a median age of 67 years, yielding an incidence of 7.4%. At that point of the pandemic RT-PCR tests were routinely performed in symptomatic patients, whereas asymptomatic cases were tested only in case of contact with a person with COVID-19.

Median age of the infected subjects was 76.0 years (IQR 69.0–82.8); 23 (41.1%) were female, 33 (58.9%) were male. Mean dialysis vintage was 37.5 months (IQR 18.3–93.0) with diabetic nephropathy being the most frequent cause of ESRD followed by nephrosclerosis, glomerulonephritis, and cardiorenal syndrome. 76.8%/ Seventy-six point eight%??? Jaya-depending on journal policy for beginning sentences with numbers? of the COVID-19 population was hypertensive, 37.5% suffered from coronary artery disease, and 44.6% were diabetic. The renal diseases and comorbidities are summarized in Table 1.

**Table 1:** Renal diseases and comorbidities of the study population and their association with mortality

	Patients positive for COVID 19	Deceased patients vs. survivors (chi-squared test)			Association with mortality in univariate analysis	
		Deceased (n = 15)	Survivors (n = 41)	*p*	OR (95% CI)	95% CI
Cause of ESRD						
Diabetic nephropathy	15 (26.8%)	2	13	0.17	0.331	0.065–1.687
Hypertension/nephrosclerosis	11 (19.6%)	4	7	0.42	1.766	0.434–7.191
Glomerulonephritis	7 (12.5%)	0	7	0.09	0.829	0.722–0.953
Cystic kidney disease	4 (7.1%)	2	2	0.28	3.000	0.083–23.491
Cardiorenal syndrome	6 (10.7%)	5	1	**0.001**	20.000	2.095–190.913
Others	13 (23.2%)	2	11	0.29	0.420	0.081–2.166
Comorbidities						
Hypertension	43 (76.8%)	9	34	0.07	0.309	0.083–1.150
Diabetes	25 (44.6%)	7	18	0.85	1.118	0.341–3.665
Coronary artery disease	21 (37.5%)	5	16	0.70	0.781	0.225–2.709
Need for immunosuppression	5 (8.9%)	3	2	0.48	1.949	0.292–12.987

Value in bold is significant (*p* < 0.05)

The overall hemodialysis population of the five centers comprised 755 patients yielding a COVID-19 incidence of 7.4% in the observation period

*ESRD* End stage renal disease, *OR* odds ratio, *CI* confidence interval

**Fig. 1:**
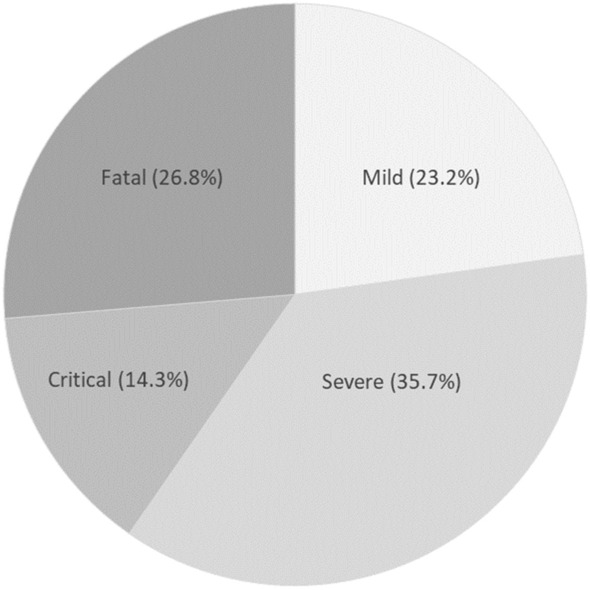
Clinical course of COVID-19 in the population of hemodialysis patients

The most frequent symptom of COVID-19 was fever (n = 31, 55.4%), followed by cough (n = 26, 46.4%). Data on diarrhea and anosmia/dysgeusia were not available for the overall study population. Among those with available data, diarrhea occurred in 19.6% and anosmia/dysgeusia in 13.5%. In 13 patients (23.2%), the disease was successfully managed in an outpatient setting (mild course). Hospitalization was necessary in 43 patients (76.8%). Of these hospitalized patients, 16 (28.6%) were transferred to the intensive care unit. 15/Fifteen—Jaya—depending on journal policy for beginning sentences with numbers? patients died from COVID-19. Thus, 23.2% showed a mild, 35.7% a severe, 14.3% a critical, and 26.8% a fatal course (Fig. 1).

Patients who died after infection with SARS-CoV-2 had a median age of 77 years (IQR 72–85) and were predominantly male (n = 11, 73.3%). Cardiorenal syndrome was associated with a significantly increased risk of mortality in a univariate analysis (OR 20, 95% CI 2.095–190.913), whereas glomerulonephritis was associated with a slightly decreased risk (OR 0.829, 0.722–0.953). Due to the low number of deceased patients, confidence intervals were large. Among the six patients with cardiorenal syndrome, five died. None of the comorbidities including hypertension, diabetes, coronary artery disease, or need for immunosuppression showed a significant association with mortality. Hypertension, however, tended to be inversely associated with death (*p* = 0.07). Accordingly, mortality was lower in those subjects with (20.9%) than without hypertension (46.1%).

In this multicenter cohort of hemodialysis patients with COVID-19, severe courses and mortality were substantially increased compared to the general population affected by COVID-19 in Germany. Whereas in this country the course was mild in > 80% of the general population [8], almost 80% of our hemodialysis population showed a severe to fatal course, with an “inverse distribution” of COVID-19 severity in the present hemodialysis patients as compared to the general population. Beyond ESRD itself, age and comorbidities are likely to contribute to this finding. Among hemodialysis patients in our series, those with cardiorenal syndrome displayed the highest risk for death due to COVID-19.

While the small number of cases requires further confirmation, it may be mentioned that our patients suffered from cardiorenal syndrome type 2; these patients overall have an extremely poor prognosis and suffer from resistance to diuretics. One-year mortality of patients with cardiorenal syndrome and moderate to severe kidney function impairment is approximately 50% [9]. Due to the limited cardiac reserve, blood pressure frequently turns from hyper- to hypotension, and this phenomenon may explain the finding of an inverse association of hypertension with fatal outcome.

The present analysis confirms that also in Germany, a country initially spared by the COVID-19 epidemic, the clinical course of COVID-19 is substantially more severe in hemodialysis patients than in the general population, and only 23% of the dialysis patients have a mild course of the disease. Among this high risk group, patients with cardiorenal syndrome and decreased blood pressure have the highest risk of death. Nephrologists should be aware of these findings in order to guarantee extended testing and early hospitalization in case of symptoms of COVID-19 pneumonia.
